# Perceived consequences of ageing with late effects of polio and strategies for managing daily life: a qualitative study

**DOI:** 10.1186/s12877-017-0563-8

**Published:** 2017-08-09

**Authors:** Catharina Sjödahl Hammarlund, Jan Lexell, Christina Brogårdh

**Affiliations:** 1 Department of Health Sciences, Lund University, Box 157, SE-221 00 Lund, Sweden; 2 The PRO-CARE Group, School of Health and Society, Kristianstad University, Kristianstad, Sweden; 3 Department of Neurology and Rehabilitation Medicine, Skåne University Hospital, Lund, Sweden; 4 Department of Health Science, Luleå University of Technology, Luleå, Sweden

**Keywords:** Activities of daily living, Aging, Emotions, Postpoliomyelitis syndrome, Psychological adaptation, Qualitative research, Self-care

## Abstract

**Background:**

New or increased impairments may develop several decades after an acute poliomyelitis infection. These new symptoms, commonly referred to as late effects of polio (LEoP), are characterised by muscular weakness and fatigue, generalised fatigue, pain at rest or during activities and cold intolerance. Growing older with LEoP may lead to increased activity limitations and participation restrictions, but there is limited knowledge of how these persons perceive the practical and psychological consequences of ageing with LEoP and what strategies they use in daily life. The aim of this qualitative study was therefore to explore how ageing people with LEoP perceive the their situation and what strategies they use for managing daily life.

**Methods:**

Seven women and seven men (mean age 70 years) were interviewed. They all had a confirmed history of acute poliomyelitis and new impairments after a stable period of at least 15 years. Data were transcribed verbatim and analysed using systematic text condensation.

**Results:**

The latent analysis resulted in three categories ‘Various consequences of ageing with LEoP’, ‘Limitations in everyday activities and participation restrictions’, and ‘Strategies for managing daily life when ageing with LEoP’ and 12 subcategories. The new impairments led to decreased physical and mental health. The participants perceived difficulties in performing everyday activities such as managing work, doing chores, partaking in recreational activities and participating in social events, thereby experiencing emotional and psychological distress. They managed to find strategies that mitigated their worries and upheld their self-confidence, for example finding practical solutions, making social comparisons, minimising, and avoidance.

**Conclusion:**

Ageing with LEoP affected daily life to a great extent. The participants experienced considerable impact of the new and increased impairments on their life situation. Consequently, their ability to participate in various social activities also became restricted. Social comparisons and practical solutions are strategies that facilitate adaptation and acceptance of the new situation due to LEoP. This emphasises the need to design rehabilitation interventions that focus on coping, empowerment and self-management for people ageing with LEoP.

## Background

Up to 80% of people who had contracted poliomyelitis in their childhood develop new or increased impairments several decades after the initial infection, commonly referred to as late effects of polio (LEoP) or postpolio syndrome (PPS) [1]. The new symptoms are characterised by muscular weakness and fatigue, generalised fatigue, pain at rest or during activities and cold intolerance [2–4]. The impairments can limit the ability to perform everyday activities [5], especially those related to standing, walking and climbing stairs [6], which increase the risk of falling [7]. This, in turn, may impact on the persons’ life satisfaction negatively [8].

Growing old with LEoP and experiencing the increased impairments, activity limitations and participation restrictions may present a challenge when the disability interacts with the effects of normal ageing [9]. Studies involving the general population have shown that ageing is time-dependent and comprises two interacting processes [10, 11]. Primary ageing involves time-dependent biological processes leading to a progressive decline of physiological functions [11]. Secondary ageing includes environmental and external factors, e.g. disease-related influences, lack of physical activity, inadequate nutrition, smoking, alcohol abuse, and stress [10].

Among people ageing with LEoP, the chances of living a healthy life in terms of physical activities may be reduced by the increased disability [12]. In addition, psychological factors, such as feeling ashamed or uncomfortable, fear of injuries, lack of motivation and energy, together with environmental factors are perceived as barriers for physical activities and participation [13–15]. Overall, this may lead to an uncontrolled downward spiral during which these individuals are in need of rehabilitation. A qualitative study revealed that persons with LEoP can benefit from an individualised, goal-oriented, comprehensive interdisciplinary rehabilitation programme [16]. The focus of the programme was to reduce self-perceived disability by providing lectures about LEoP, presenting self-management strategies and a variety of interventions to maximise each individual’s physical, mental and social potential. The participants described the rehabilitation programme as the start of a process whereby they acquired new skills, which, over time contributed to a good but different life. Approximately a year after the programme the participants described that they had acquired a sense of control, accepted life with LEoP, established new habits, had taken on a changed valued self and could look to the future with confidence [16]. Thus, rehabilitation can have a profound effect on daily life as people with LEoP grow older. However, our understanding of how these persons are managing ageing and what strategies they use to overcome the difficulties are not yet fully understood. According to Folkman and Lazarus [17, 18] both problem-focused and emotion-focused strategies are often used in order to increase functioning and to mitigate stress and worry.

To the best of our knowledge, only one study [19] has qualitatively investigated how the consequences of ageing with LEoP are perceived. That study described that the physical impairments were related to emotional distress. However, it did not describe which strategies were used to manage the practical and psychological consequences of ageing with the sequaele of polio. As many persons with LEoP are ageing with a disability that can affect daily life, a deeper understanding is needed to guide clinicians in designing more individually targeted rehabilitation interventions.

The aim of this study was therefore to explore how ageing people with LEoP perceive the consequences of ageing and the strategies they use to manage daily life.

## Methods

### Research design

Qualitative research design with individual interviews was used, as it allows us to explore an insider’s perspective of the phenomenon. The interviews included questions about different aspects of the participants’ experiences of growing up, and later in life when ageing with LEoP, as well as falls and fear of falling [20]. In this study, only data from the interviews related to the participants’ experiences of ageing are presented. Systematic text condensation was used to analyse the data, which is a descriptive and explorative method inspired by phenomenological theory [21].

### Participants

A total of 14 participants (7 women and 7 men) volunteered for the study. All of them had a confirmed history of acute poliomyelitis with new symptoms after a stable period of at least 15 years and a clinically verified LEoP. They were strategically selected from a rehabilitation clinic in the south of Sweden with regard to gender, age and time since the onset of LEoP, and current disability [22]. Their mean age was 70 (range 61–78) years and the mean duration of LEoP was 26 years (range 9–43). Eleven participants lived with a partner, four were working and ten had part-time disability pension or old age pension (Table 1).

**Table 1:** Characteristics of the 14 participants with late effects of polio (LEoP)

Age; mean years (min-max)	70 (61–78)
Gender (men/women), n	7/7
Age at onset of acute polio; mean years (min-max)	4 (1–12)
Duration of late effects of polio; mean years (min-max)	26 (9–43)
Living situation; with a partner/alone, n	11/3
Vocational situation; working/disability pension or old age pension, n	4/10
Residential location; house/apartment, n	8/6
Self-reported impairments^a^, mean (min-max)	27 (19–39)
Walking ability, n	
< 100 m/ 100–1000 m/ > 1000 m	3/7/4
Use of mobility device, n	11

^a^Measured by the Self-reported Impairments in Persons with late effects of Polio (SIPP) rating scale, score 13–52 points, higher score = greater impairments

### Ethics

All individuals gave their written informed consent to participate after receiving detailed information about the study. The principles of the Declaration of Helsinki were followed and the study was approved by the Regional Ethical Review Board in Lund, Sweden (Dnr: 2014/186).

### Procedure

Data were collected through personal interviews (average 70 min, range 60 – 90 min) conducted by two of the authors (CB and CSH). Each interview started with an introductory sentence: ‘The aim of this interview is to better understand how persons with LEoP experience ageing and the consequences of LEoP, that may influence the life situation’. The interview guide included areas of interest such as (a) managing everyday activities in the current life situation and (b) strategies to overcome perceived difficulties. Follow-up questions such as: ‘Can you give an example?’, ‘I’m not sure what you mean’, and ‘Could you elaborate on this subject?’ were used. All interviews were recorded and transcribed verbatim.

### Data analysis

Data were analysed using systematic text condensation (STC) as described by Malterud [21]. First, the interview transcripts were read to get a general impression of the whole and to identify and categorise the primary themes. Next, meaning units were identified and formulated into codes representing the essence and meaning of the statements. During this phase, two of the authors (CB, CSH) worked individually to uncover more perspectives and nuances of associations in the material. Subsequently, both authors merged the coded data into one set of data. The coded data were then organised by their conceptual representation into subcategories and duplicates were removed. Following this, the content of the meaning units of each category was reviewed, and the gist was formulated into aspects representing the content. The subcategories were then organised into categories. To validate the categories and make sure that no essential aspects had been overlooked, the clusters were referred back to the raw data, and read through once again by the authors. Finally, the re-contextualised data were formulated as conceptual descriptions of the meaning of each category and representative quotes were selected for each category/subcategory.

## Results

**Fig. 1:**
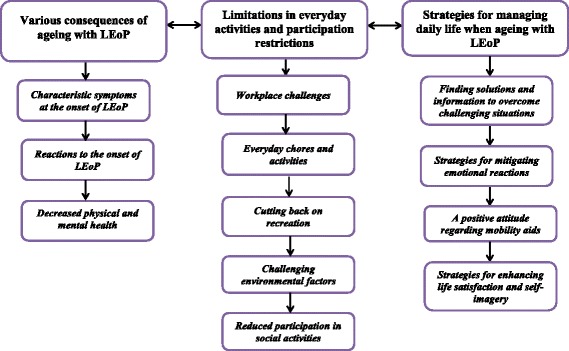
The elaborated categories and subcategories of how ageing persons with late effects of polio (LEoP) perceived the consequences of ageing and the strategies used to manage daily life. The arrows illustrate that they are interrelated

The latent analysis of the elaborated subcategories gave rise to three categories: 1) ‘Various consequences of ageing with LEoP’ with three subcategories (a) Characteristic symptoms at the onset of LEoP, (b) Reactions to the onset of LEoP, and (c) Decreased physical and mental health; 2) ‘Limitations in everyday activities and participation restrictions’ with five subcategories (a) Workplace challenges, (b) Everyday chores and activities, (c) Cutting back on recreation, (d) Challenging environmental factors, and (e) Reduced participation in social activities; and 3) ‘Strategies for managing daily life when ageing with LEoP’ with four subcategories (a) Finding solutions and information to overcome challenging situations, (b) Strategies for mitigating emotional reactions, (c) A positive attitude regarding mobility aids, and (d) Strategies for enhancing life satisfaction and self-imagery (Fig. 1).

### Various consequences of ageing with LEoP

#### Characteristic symptoms at the onset of LEoP

The experiences of the onset of LEoP differed. Some perceived a gradual increased tiredness and fatigue, which was both physical and mental. Others felt that these changes were more sudden and unexpected, as if all their strength and energy had vanished.

*Tiredness… both physical and mental, but perhaps more physical... For some reason, the body just stopped working, it wasn’t working any more.* 10 J18.

The body felt as if it had collapsed and it could not be managed or controlled due to a sudden weakness, which made it impossible to even stand up. Balance was noticeably impaired and the participants felt increased muscular pain. Some participants did not even know that LEoP existed and therefore did not understand what was happening.

*Well, no… I was really down and depressed… and, well I always want to be the big achiever, although I really should have asked for help… And although I really needed help I thought I could fix it on my own, but I couldn’t…I think it was all ‘cause I didn’t know why I got so exhausted... and my legs, they wouldn’t cooperate and I was tired… really tired and in pain…* 12 L24.

#### Reactions to the onset of LEoP

Initially, the participants were concerned about their body malfunctioning. They were not prepared for having the same symptoms as during the polio infection in their childhood, which they thought they had overcome.

*Well, if you put it like this... when you have recovered to the extent as I have... I mean, I could go out dancing and I... and then… then… well, you didn’t expect something like this to happen afterwards, that something like this could occur like now with the late effects of polio. You thought that… well, you just didn’t expect that…* 14 N11.

Their reactions showed surprise, frustration and anger that the original disease would affect their lives negatively once more. There was also some confusion as to which of the symptoms were due to LEoP or just normal ageing.

*It’s a little hard to say, I′ve grown old in the meantime too, so what is the post-polio, and what are the symptoms of aging? It’s all a little confusing.* 1A2.

#### Decreased physical and mental health

All participants described that they could not manage as well as they did before the onset of LEoP. They felt weaker and became physically tired more quickly. Pain was another symptom that had an impact on their physical ability, and also affected their mood and sapped a lot of energy. Some participants became physically fatigued and breathless more easily when they exerted themselves. These increased impairments affected their walking ability and mobility related activities.

*Well, as soon as I want to do something physical then… then I almost always get muscle soreness... if I compare with my younger days, I hardly ever had sore muscles, regardless of what I was doing, because then you were sort of more active all the time…* 6F3.

Most of the participants also reported that stress was more difficult to handle than before and that they worried, for example, about not being able to be on time. They also reported that they suffered a different kind of fatigue not related to their physical effort. This was described as an extreme tiredness that consumed all their energy.

*Before you could be tired because you exerted yourself… and you felt that your body was tired. But this tiredness takes all of me and not just the body... it’s strange. It somehow drains your energy completely.* 5E23.

The participants could no longer rely on their less affected limb, as they had done after the poliomyelitis infection. They felt that they had previously been just as physically active as their friends, and they were not fully prepared for the new consequences.

*…all my weight was carried by the left* (less affected) *leg. When riding the bike, the left leg was doing all the work, the right leg was just following along so to speak… it had no muscles…. you see, that leg isn’t functioning and therefore the left leg has taken all of the strain.* 10 J3.

### Limitations in everyday activities and participation restrictions

#### Workplace challenges

Managing their jobs became more and more exhausting. The participants noticed that they had difficulty in tolerating the workload. Some of them changed to different jobs that were less physically demanding, whereas others had to shorten their working hours. They said that this kind of change made them feel as though they were no longer good enough for their jobs, which was hard to accept. The participants also felt that they missed out on the social perspectives of their work life.

*Then you had to cut off some of the work time. It was annoying too. You had turned into three-quarters of a human, and then you became only half a person… I had a hard time accepting that. I liked what I was doing.* 10 J18.

#### Everyday chores and activities

Household chores were also perceived as exhausting. Especially cleaning, vacuuming and shopping caused a lot of pain, but at the same time these activities had to be done. Taking a shower and getting dressed were more time consuming than before and entailed movements which were difficult to do, for example balancing and standing on one leg.

.*..well, some things are causing me a lot more pain afterwards, you know… for example vacuuming… oh, that’s pure agony, and then you’re in pain for two days. But it still needs to be vacuumed, you know.* 1A6.

*The hardest situation in my everyday life is showering… well before I used to take a shower every day or every second day, but now I shower every fourth or fifth day or... I can’t stop doing it, but I sense quite a lot of discomfort every time I take a shower because it’s really hard…* 6F4.

#### Cutting back on recreation

The participants stated that they had become much more physically inactive and unable to partake in recreational activities than they did in their younger days. Now, their increasing disability forced them to cut back on recreation. Above all, they missed being able to to ride a bicycle or go for a walk. Some also had to give up activities involving the upper limbs, e.g. to embroider or paint.

*…I would like to walk more, I would like to be able to run, but I can’t. Biking, also. I used to ride my bike very much before, but now I don’t have that strength in my legs. It’s okay on flat ground, but not otherwise. I have also a hard time stopping the bike to get off, I’ve fallen off a couple of times. I don’t have the balance I need…..* 7G6.

#### Challenging environmental factors

The participants felt increasingly vulnerable to environmental factors that were obstructive or limiting, for instance, irregularities on sidewalks and streets, bad lighting at night and bad weather conditions like a strong wind or snow and ice. Means of transportation that were not adapted for people with disabilities were also aggravating.

*I used to drive down to swim in the sea. I can’t do that anymore because you have to walk down steps and I don’t know if I can come up again.* 8H10.

#### Reduced participation in social activities

The increased impairments made it hard to make plans to meet with family and friends. To sit down for a long period of time was hard due to low back pain. To go out with friends and join in activities which were enjoyed earlier had also become increasingly difficult, for example going to the movies, theatres or dinner parties.

*… you have to say no to a lot of invitations, for example going to the movies or the theatre. I can’t go to the movies or such like, I can’t sit still for that long because of my back. Unfortunately… that’s also something which is new and it’s hard to deal with.* 10 J24.

Although they could join in different outings, they were unable to walk more than short distances because of getting very tired. Poor balance and walking ability also made it difficult to walk amongst crowds.

*If I’m out walking with other people I can’t walk at the same pace as they do… If we’re a group of people walking together, we may split up so that some walk at the same speed as I do, and the others walk a bit faster. That’s one kind of situation that I feel has changed.* 4D14.

### Strategies for managing daily life when ageing with LEoP

#### Finding solutions and information to overcome challenging situations

Some strategies were focused on finding practical solutions, but also information and explanations were sought for the increased disability following LEoP. These strategies included planning every move in challenging situations, employing hired help for heavy work around the house and garden, taking regular breaks or resting, using different forms of physical training, and stretching to maintain physical capacity.

*I don’t avoid taking part in physical activities, but I really can’t do everything, you have to choose to do the things that you can manage… also when working in the garden you need to take more breaks than before.* 7G5.

#### Strategies for mitigating emotional reactions

Strategies to mitigate the psychological consequences included comparing what others in the same situation were doing, trying to think positively, avoiding thinking about the problems, and making comparisons with those who seemed to manage better than they did, e.g. to assume that they managed just as well as healthy elderly people of the same age.

*But on the other hand, if I were healthy maybe I couldn’t have done it to the full anyway… you don’t know that. You have to have this sort of perspective to be able to deal with it.* 7G9.

To meet others with LEoP and participate in the postpolio advocacy groups involved both positive and negative aspects.

*… anyway, you saw people who were worse off than me, you know. So I thought that I don’t need to be moaning about it because…* (laughs*)... well… There are those who are in a worse situation…* 14 N17.

#### A positive attitude regarding mobility aids

In their earlier days the participants described how they had been reluctant to use aids. It made them look different compared to their peers which was awkward. Now, they felt more comfortable and safe using mobility aids and that they were functional and necessary in managing everyday activities. Using an electric wheelchair also gave a sense of freedom and participation.

*I was surprised myself at how much I actually am using it* (the electric wheelchair)*, actually it’s quite a lot more than I thought at the beginning... and I feel much less restricted with it.* 2B7.

#### Strategies for enhancing life satisfaction and self-imagery

Although the participants had lived with their disability for many years, they felt that they had had a good life and been able to do most of what they wanted to do. They felt fortunate that they had been able to live a life which was similar to those who were able-bodied.

*…but in all other respects I feel fortunate. I have been able to live a normal life. It’s not until recently that there have been changes, but that may well be a combination of getting older as well…* 5E14.

After the onset of LEoP, most of the participants described how their self-image had changed. After recovering from polio, they had struggled to be on a par with those who were healthy and not see themselves as disabled. In that sense they felt that LEoP had changed the way they looked at themselves.

*Actually, I have never been in any way aware, in my everyday life or any other circumstances, that I am disabled until... well I don’t know... about five years ago or something. I mean, that’s when I started to have pain, everything went really slow, it was never finished, aaahh, one didn’t have the strength. One couldn’t keep up, you know…* 1A6.

## Discussion

The main findings of this qualitative study were that ageing persons with LEoP experienced considerable impact caused by the new and increased impairments to their health and ability to perform daily activities. They were no longer able to take part in various social activities, which was hard to accept. Although emotional and psychological distress were present, the participants managed to find strategies that mitigated their worries and supported their self-confidence. Social comparisons, minimising and practical solutions were strategies that facilitated their adaptation to and acceptance of their new situation with LEoP.

### The consequences of ageing with LEoP

The increased impairments made it difficult to live the life they were used to, and they needed to cut back on activities and social events. A major challenge was that the increased impairments also brought back memories of the original traumatic experiences associated with contracting polio in their childhood. Throughout the interviews, sentiments of frustration, disappointment and feeling mixed-up were central. Regardless as to whether the onset of LEoP was sudden or gradual, the participants were not prepared for the impact it had on their daily life. Some decided to ignore these symptoms and continued as before, a strategy that had been successful in their youth and adulthood [23]. The initial psychological reactions included worrying and denying, which have been reported in earlier studies [24–26]. The psychological challenges when facing LEoP and the psychological reactions meant redefining themselves and their life situation, which was also line with these studies [24–26]. The physical manifestations also resulted in increased vulnerability to environmental factors such as bad weather, stairs, uneven sidewalks and similar factors, all of which also affected their ability to engage in activities and participate in social events [27]. Social interactions and being able to engage in activities related to household management and recreational pursuits were important for their life satisfaction, which is in agreement with the findings of a previous study [28].

### Managing the limitations in daily activities and participation restrictions

Not being able to work was one aspect that was disappointing. It was important to uphold social norms of being financially independent and providing for yourself and your family, which has been described previously [26, 29]. To be equal to those who were healthy and to blend in whilst struggling to overcome obstacles were strategies that had guided them since childhood [24, 30]. This may explain why they felt overwhelmed by LEoP as their earlier problem-focused strategies no longer worked.

To manage the psychological consequences of their disability they used strategies aimed at simplifying the way in which they adapted to the new situation, and to reduce their anxiety and psychological stress. A problem-focused and active coping strategy may be effective if used when the individual feels that the problem can be solved in a positive way or if the stimuli causing the distress may be neutralised, e.g. organising and planning activities in daily life, and actively seeking information and support [17, 18]. Emotion-focused coping, e.g. wishful thinking and avoidance, may be of use if the individual’s psychological defences are no longer able to maintain an emotional balance or to handle the emotional stress of anxiety and depression [17, 18]. In the present study, there was a shift from problem-focusing towards emotion-focusing strategies compared to findings in previous studies of persons with LEoP [5, 14, 30, 31]. One explanation may be that the participants in the present study were older compared to the previous studies and that their problem-focused strategies were no longer effective. The gap between their physical functioning and the environmental demands had increased further, which has also been described in a previous study [14].

The physical impairments affected their ability to perform everyday activities. Among the household chores, vacuuming was one of the most dreaded and exhausting activities. Other activities that had become hard to do, such as work around the house and gardening, were managed by hired help. As this was not unusual in the society at large, this problem-focused strategy was relatively easy to accept. Personal care was difficult to manage, for example taking a shower or getting dressed. Not being able to care for your personal hygiene was an emotional stressor and caused fear of losing one’s independence [14, 25, 26]. A major challenge, when trying to manage the new or re-occurring symptoms, was that the participants no longer could rely on the less affected limb as compensation. Most participants suffered from overuse and strain that they had forced on themselves in order to maintain a normal life.

A central theme was that the participants stated that their increased impairments were or could be caused by normal ageing. They compared their symptoms of physical deterioration and environmental challenges to community-dwelling adults about the same age. Strategy using social comparisons may come from the participants’ urge to be just as capable as anyone else [26]. By blaming their impariments on normal ageing, they were no different from other persons who were getting older, which may have reduced some worry. The feeling of belonging to a group by claiming that “we’re all in the same boat” may reduce anxiety [32], and this strategy is also in line with their childhood strivings [30, 31]. Furthermore, the use of social comparisons and minimising may have facilitated the use of mobility aids to compensate for the deteriorating functioning. We also found a more positive attitude towards mobility aids compared to a previous study [5]. One explanation may be that the participants were now getting older and that older adults of a similar age were also using mobility aids.

Previous studies [24, 26, 31] have shown that people with LEoP, who had ‘invisible symptoms’, were among those who found it most difficult to accept deteriorating functioning. This was also present in our results. Those who expressed most difficulty in accepting LEoP had once mastered the same challenges as their healthy peers in sports or even when dancing in high-heeled shoes. Another strategy to reduce distress, apart from social comparisons and minimising, was avoidance, which has been reported previously [5, 19, 33]. Avoidance, which is an emotion-focused strategy, may prevent more active, problem-focused strategies, and this needs to be considered when designing rehabilitation interventions.

The results of our study highlight important and complex aspects of how LEoP is interwoven with normal ageing. In a previous study [16], people with LEoP expressed that a sense of control, implementing new habits, and accepting life with new symptoms was an important part of having a positive attitude and looking to the future with confidence. The central role of emotional and psychological reactions, as seen in the present study, add to these results and will possibly have important implications for the design of future rehabilitation interventions.

### Methodological considerations

The participants were strategically selected and covered a wide range of variations in age, clinical symptoms and years of experiencing LEoP. During the data collection a semi-structured interview-guide was used to ensure that all areas were covered. Fourteen participants were included as no new information was collected in the last interviews. The participants provided rich and relevant data, which gave valuable insights into the impact of ageing with LEoP. However, as the participants suffered from mild to moderate impairments the results cannot be fully representative of the entire population of persons with LEoP.

Throughout the data analysis reflexivity has been considered, i.e., we have been aware that the preunderstanding that the authors may have as clinicians and researchers could contaminate the data, if one is not fully aware of previous experiences [34]. All authors worked separately during the process of condensing the data to meaning units and coding. There were continuous discussions during the analysis aimed at keeping us aware that our decisions might be influenced by our previous experiences. This helped us to stay neutral as to the data. STC was chosen as a suitable qualitative method as the procedure facilitated cross-case synthesis amongst the participants. In addition, we have also presented the participant’s code number after each quotation to show the representation of our participants, and to add transparency and trustworthiness to our findings and interpretations of the data.

## Conclusions

Participating ageing persons with LEoP experienced a considerable impact of the new and increased impairments on their life situation and on their ability to perform daily activities. Consequently, their capacity to participate in various social activities had become restricted. Although emotional and psychological distress were present, the participants managed to find strategies that mitigated their worries and supported their self-confidence. Social comparisons, minimising and practical solutions were strategies that facilitated their adaptation and acceptance of their new situation with LEoP. This emphasises the need to design rehabilitation interventions for ageing people with LEoP that focus on coping, empowerment and self-management.

## Abbreviations

LEoP: late effects of polio
PPS: Postpolio syndrome
